# Identification of Abnormal Biliary Anatomy Utilizing Real-Time Near-Infrared Cholangiography: A Report of Two Cases

**DOI:** 10.1155/2017/8628206

**Published:** 2017-04-27

**Authors:** Joseph Bozzay, Diego Vicente, Elliot M. Jessie, Carlos J. Rodriguez

**Affiliations:** Walter Reed National Military Medical Center, 8901 Rockville Pike, Bethesda, MD 20889, USA

## Abstract

Biliary duct anomalies are commonly encountered during laparoscopic cholecystectomy. Advancements in the field of surgery allow for enhanced intraoperative detection of these abnormalities. Fluorophore injection and near-infrared (NIR) imaging can provide real-time intraoperative anatomic feedback without intraoperative delays and ionizing radiation. This report details two cases where the PINPOINT Endoscopic Fluorescence Imaging System (NOVADAQ, Ontario, Canada) was used to identify anomalies of the biliary tree and guide operative decision-making.

## 1. Introduction

The laparoscopic cholecystectomy is one of the most common procedures performed by general surgeons today [1]. Anomalous biliary and arterial anatomy can be encountered in up to 50% of cases and can lead to intraoperative challenges, particularly in patients with significant inflammatory changes [2]. Traditionally, intraoperative cholangiography (IOC) has been very useful for the detection of such biliary tree anomalies [1, 3]. However, IOC requires additional participants in the operative procedure, exposes the patient and operative team to ionizing radiation, and can prolong the case [1, 3].

Fluorescent cholangiography (FC) is a technique that can be easily performed with intravenous fluorophore injection and intraoperative near-infrared (NIR) imaging to view its dissemination throughout the biliary system [1, 4, 5]. This allows for real-time feedback during the operation, thereby eliminating logistics associated with performing IOC. There are multiple systems available, including PINPOINT Endoscopic Fluorescence Imaging System (NOVADAQ, Ontario, Canada), the NIR/ICG system (KARL STORZ, Tuttlingen, Germany), and the STRYKER Infrared Fluorescence (IRF) Imaging System (STRYKER Endoscopy, San Jose, CA). Our institution owns a PINPOINT system which was used for our NIR FC. We present two cases where this technology was utilized to identify anomalous biliary and arterial anatomy in patients undergoing laparoscopic cholecystectomy. In addition to summarizing the basics of NIR FC, this report will also discuss NIR FC clinical trials and the various surgical applications of NIR.

## 2. Case Report 1

Our first patient was a 29-year-old female with a recent episode of gallstone pancreatitis without ultrasound evidence of cholecystitis. An indeterminate preoperative MRCP failed to rule out retained stones in the biliary tree; therefore, IOC was planned. Her past medical history was notable for familial adenomatous polyposis (FAP) and primary hyperparathyroidism. Her past surgical history included a parathyroidectomy, appendectomy, total proctocolectomy with ileal pouch-anal anastomosis and protective ileostomy, reversal of ileostomy, and laparoscopic ventral hernia repair with intra-abdominal mesh placement. Given her multiple intra-abdominal surgeries, ventral mesh, and recent medical history, she was considered a potential challenging laparoscopic case and it was felt that PINPOINT NIR could offer intraoperative assistance and guidance for the expected dissection.

The patient was injected with 1 mL (2.5 mg) of indocyanine green (ICG) at anesthesia induction. In order to avoid trocar placement through the mesh, a 12 mm trocar was placed via direct visualization in the left upper quadrant, just off the tip of the 11th rib; two 5 mm trocars were placed in the usual subcostal position of the right upper quadrant; and one 5 mm trocar was placed midline, in the subxiphoid position. Eventually, a 10 mm trocar was placed lateral to the mesh, in the left lower quadrant for improved visualization of the gallbladder.

**Figure 1:**
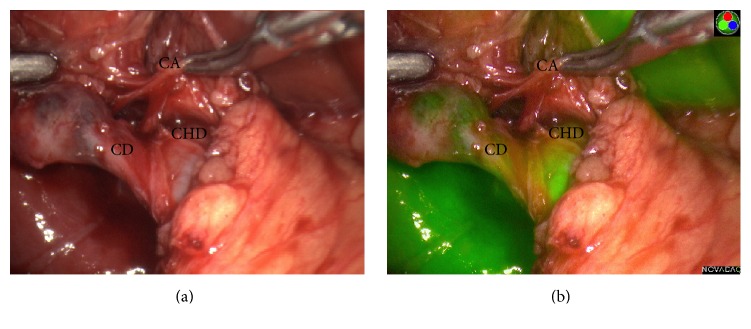
White light view (panel a) and PINPOINT view (panel b) of the laparoscopic cholecystectomy in the patient with a short cystic duct. CA: cystic artery, CHD: common hepatic duct, and CD: cystic duct.

A 10 mm 30° PINPOINT NIR equipped laparoscope was placed through the 12 mm port to provide visualization of the biliary tree. Dense adhesions between small bowel, liver edge, and gallbladder were encountered during dissection to expose the gallbladder. As dissection proceeded towards the infundibulum, FC was periodically performed by selecting the PINPOINT NIR mode on the camera. This allowed us to quickly switch between white light mode and NIR mode (Figures 1(a) and 1(b)). FC easily visualized the gallbladder, a shortened cystic duct, and the common hepatic/cystic duct confluence.

FC allowed for early detection of the confluence of the cystic and common hepatic duct, thus enabling safe, blunt dissection of Calot's Triangle in the setting of a shortened cystic duct. This confluence was not easily seen with the regular white light mode (Figure 1(a)).

After performing a preoperatively planned IOC which was unremarkable, we proceeded to divide the cystic duct. However, we did not feel that the duct was of adequate length for three clips as we were concerned that the distal clip might entrain the biliary tree confluence leading to stricture. Two clips were placed on the cystic duct and two were placed on the infundibulum in order to prevent bile spillage during removal of the gallbladder from the fossa. The gallbladder was removed without difficulty.

On pathologic evaluation, the gallbladder did have evidence of chronic cholecystitis and contained multiple gallstones. The patient recovered without incident from the surgery and was discharged from the hospital the next day. She was seen in clinic and had no complaints. In this case, FC allowed for early identification and safe dissection of a short cystic duct in a challenging patient requiring extensive dissection associated with prior surgery and inflammatory changes from acute pancreatitis and chronic cholecystitis.

## 3. Case Report 2

Our second patient was a 71-year-old female who had just undergone an endoscopic retrograde cholangiopancreatogram (ERCP) extraction of a common bile duct stone following an episode of gallstone pancreatitis. Her past medical history was notable for paroxysmal atrial fibrillation, but she was otherwise healthy.

As with the first patient, this patient received 1 mL (2.5 mg) of IV ICG at the time general anesthesia was induced. Standard Hasan infraumbilical, subxiphoid (1–5 mm), and RUQ (2–5 mm) ports were placed. Significant adhesions between the liver and abdominal wall were present which suggested Fitz-Hugh-Curtis disease. Once these adhesions were released, the fundus was identified, grasped, and retracted in a cephalad manner.

**Figure 2:**
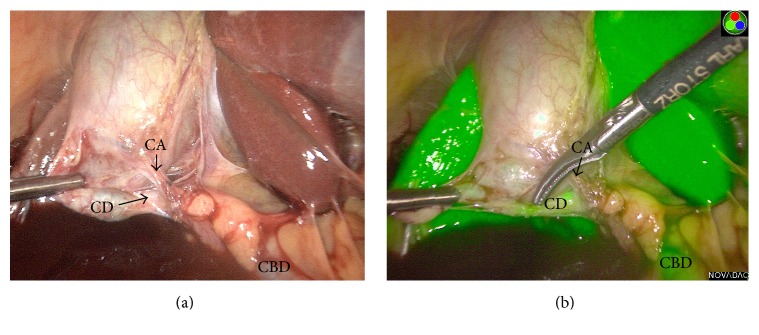
White light view (panel a) and PINPOINT view (panel b) of the laparoscopic cholecystectomy in the patient with a long cystic duct and an anterior cystic artery. CA: cystic artery, CBD: common bile duct, and CD: cystic duct.

Dissection of the critical structures revealed the cystic artery to be anterior to the cystic duct with origination from either the proper or right hepatic artery. PINPOINT NIR confirmed that the cystic duct coursed posterior to the cystic artery (Figure 2) and aided in direct visualization of abnormal anatomy. The cystic artery and cystic duct were ligated with clips, divided in standard fashion, and the gallbladder was removed.

The patient was discharged from the hospital without incident on the first postoperative day.

## 4. Discussion

PINPOINT is the laparoscopic version of the open SPY system. There are other fluorescent cholangiography systems including the NIR/ICG system from KARL STORZ and the STRYKER Infrared Fluorescence (IRF) Imaging System. It utilizes a white light camera and an NIR fluorescence excitation and acquisition system with ICG as the fluorophore [4, 6]. Through pressing a camera-mounted button, PINPOINT can display simultaneous video modes to include conventional white light high definition, fluorescence only, and composite NIR-ICG overlay modes [4–6]. ICG binds to plasma proteins after IV injection, thus enabling it to remain within the intravascular system; however, it only remains in the intravascular space for about three minutes [4–6]. Bound ICG is readily taken up by the liver and is then excreted, unchanged, through the biliary system [1, 5, 7]. ICG is excreted into the bile within minutes after injection and reaches peak concentration at about two hours; however, preoperative injection allows for 30–45 minutes to reach the biliary tree in sufficient concentration to fluoresce when exposed to NIR [1]. Our institution's experience has shown with these and other cases that NIR will continue to show hepatobiliary system enhancement well over two hours after injection.

ICG injection is generally well tolerated with anaphylactic reaction being an exceedingly rare but serious risk [4, 5]. It contains no more than 5% of sodium iodide and so it should be used cautiously in patients who have an allergy to iodides or iodinated imaging agents [4, 5].

Real-time NIR imaging has proven applications to biliary surgery. Matsui and colleagues found that NIR florescence allowed for good identification of biliary anatomy during open or laparoscopic surgery, and they were able to immediately identify damaged or constricted ducts. They concluded that NIR FC provides sensitive and prolonged identification of biliary anatomy and assessment of functional status [7]. A case report with video shows active PINPOINT use to identify an anomalous duct which was thought to be an aberrant right hepatic duct [8]. This helped direct appropriate management of the cystic duct and the patient had no complications [8].

Hutteman and colleagues found ICG-NIR useful for visualizing the common bile duct and biliary anastomoses during pancreaticoduodenectomy and suggested that it could be beneficial in biliary cases with difficult surgical anatomy, as was apparent in our cases [9]. To further evaluate this hypothesis, NIR FC is currently the subject of a randomized study evaluating operative time and safety of operative technique (including bile duct injury and resident autonomy and identification of structures) compared to normal laparoscopic cholecystectomy methods [10]. Another clinic trial by Cleveland Clinic, Florida, has been designed to evaluate the effectiveness of NIR FC compared to standard white light for identifying extrahepatobiliary structures [11]. The multicenter FALCON trial in Netherlands is randomizing patients to either NIR FC or conventional laparoscopic imaging and comparing the time to identification of the “critical view of safety” in addition to other endpoints [12]. These ongoing clinical studies may help determine which patients will benefit the most from the use of NIR FC and if surgical outcomes are affected by its use.

In addition to biliary imaging, NIR-ICG fluorescence has widespread application in surgery to include intraoperative vascular perfusion assessment of myocutaneous flap perforators and hollow viscus anastomoses, as well as oncologic procedures [5, 13, 14]. It is extensively used in the field of plastic surgery to determine the predicted viability of myocutaneous pedicled and free flaps and for assessing skin viability in complex abdominal wall reconstruction cases [5, 13]. Additionally, a recent systemic review concluded that microperfusion techniques utilizing florescent dyes may help guide the management of colorectal anastomosis and impact postoperative complications [15]. In the recent PILLAR II study, NIR was used to intraoperatively assess anastomotic perfusion during colorectal surgery and guide the management of the anastomosis [4]. The cases that had anastomotic adjustments based on NIR perfusion assessment did not develop anastomotic leaks. Shimada et al. reported that ICG fluorescent imaging was useful for evaluating the blood supply to esophagectomy anastomoses but did not find that it reduced the rate of anastomotic leaks in their population of 40 patients [16]. Real-time fluorescence imaging is also being used in the field of surgical oncology to detect disease and improve debulking and wide local excisions and anatomically delineate surrounding structures during organ removal [14]. The ability of NIR to differentiate various tissues offers an advantage over preclinical imaging and the limitations of the human eyesight [14]. Future clinical trials will help further characterize the advantages and limitations of these approaches and determine the impact of NIR on surgical outcomes.

Advances in the field of surgery have allowed surgeons to develop new techniques and to improve operative outcomes. Obvious advantages of NIR FC include real-time anatomic biliary mapping, ease of use, and no need for additional equipment. NIR FC may prove to be a valuable asset in the operating room. Future well-designed studies, such as the one described above, will be useful in this area. Certain patient populations such as those with inflammation or tissue distortion around the gallbladder may benefit from more routine use of NIR FC systems when undergoing laparoscopic cholecystectomy.
